# No Sex Differences in Self-Reported Childhood Maltreatment in Major Depressive and Bipolar Disorders: A Retrospective Study

**DOI:** 10.3390/brainsci12060804

**Published:** 2022-06-19

**Authors:** Daniela Caldirola, Tatiana Torti, Francesco Cuniberti, Silvia Daccò, Alessandra Alciati, Koen Schruers, Giovanni Martinotti, Domenico De Berardis, Giampaolo Perna

**Affiliations:** 1 Department of Biomedical Sciences, Humanitas University, Via Rita Levi Montalcini 4, 20090 Pieve Emanuele, Italy; daniela.caldirola@hunimed.eu (D.C.); tatiana.torti@gmail.com (T.T.); francesco.cuniberti@hunimed.eu (F.C.); silvia.dacco@hunimed.eu (S.D.); giampaolo.perna@hunimed.eu (G.P.); 2 Department of Clinical Neurosciences, Villa San Benedetto Menni Hospital, Hermanas Hospitalarias, Via Roma 16, 22032 Albese con Cassano, Italy; alessandra.alciati@gmail.com; 3 Humanitas San Pio X, Personalized Medicine Center for Anxiety and Panic Disorders, Via Francesco Nava 31, 20159 Milan, Italy; 4 ASIPSE School of Cognitive-Behavioral-Therapy, Via Luigi Settembrini 2, 20124 Milan, Italy; 5 Humanitas Clinical and Research Center, IRCCS, Via Manzoni 56, 20089 Rozzano, Italy; 6 Research Institute of Mental Health and Neuroscience and Department of Psychiatry and Neuropsychology, Faculty of Health, Medicine and Life Sciences, Maastricht University, 6226 NB Maastricht, The Netherlands; koen.schruers@maastrichtuniversity.nl; 7 Department of Neuroscience, Imaging and Clinical Sciences, University “G. d’Annunzio”, 66100 Chieti, Italy; giovanni.martinotti@gmail.com; 8 Department of Mental Health, ASL 4, 64100 Teramo, Italy; 9 Head, Department of Mental Health, NHS, ASL 4 Teramo, Contrada Casalena, 64100 Teramo, Italy

**Keywords:** childhood trauma, major depressive disorder, bipolar disorder, sex difference, age

## Abstract

Background: We investigated, for the first time, whether there are any sex differences in retrospective self-reported childhood maltreatment (CM) in Italian adult patients with major depressive disorder (MDD) or bipolar disorder (BD). Furthermore, the potential impacts of patients’ age on the CM self-report were investigated. Methods: This retrospective study used the data documented in the electronic medical records of patients who were hospitalized for a 4-week psychiatric rehabilitation program. CM was assessed using the 28-item Childhood Trauma Questionnaire (CTQ), which evaluates emotional, physical, and sexual abuse, as well as emotional and physical neglect. The linear and logistic regression models were used (α = 0.01). Results: Three hundred thirty-five patients with MDD (255 women and 80 men) and 168 with BD (97 women and 71 men) were included. In both samples, considerable CM rates were identified, but no statistically significant sex differences were detected in the variety of CTQ-based CM aspects. There was a significant association, with no sex differences, between increasing patients’ age and a decreasing burden of CM. Conclusion: Both women and men with MDD or BD experienced a similar and considerable CM burden. Our findings support routine CM assessment in psychiatric clinical practice.

## 1. Introduction

Childhood maltreatment (CM) has been widely regarded as a major public health issue due to its detrimental impact on both physical and mental health. Through a cascade of changes in the development of the nervous, endocrine, and immune systems [1], CM can increase the lifelong risks for medical [1,2,3,4,5] and psychiatric illnesses, including major depressive disorder (MDD) and bipolar disorder (BD) [1,6,7,8,9]. 

CM refers to acts of commission or omission that cause actual or potential harm to the health, survival, development, or dignity of children under the age of 18; this includes, but is not limited to, childhood physical, sexual, or emotional abuse (CPA, CSA, and CEA, respectively), and childhood physical or emotional neglect (CPN and CEN, respectively) [9,10]. Recent official reports estimated that there is a rate of child abuse and neglect of 8.4 victims per 1000 children in the United States [11]; 18 to 55 million children as victims of some kind of maltreatment in Europe [12]; and a victim rate of maltreatment of 9 per 1000 children in Italy [13]. 

In population-based studies worldwide, self-reported CM was found to be highly prevalent, with approximately 30–35% of participants reporting at least one type of CM [14,15,16]. Taking into account that geographical, socioeconomic, and methodological factors can affect differences in CM estimates from around the world [17,18], the recent international prevalence of childhood sexual abuse (CSA) in the general population was approximately 13%, with a higher rate in women (approximately 18%) than in men (approximately 8–9%) [14,15,16]. Larger variations in terms of prevalence rates were identified for the other CM types, with estimates ranging from 12% to 35–40% [12,14,15]. In contrast to the widely, but not universally [17] observed female majority of sexual victimization, there did not appear to be a sex-based preponderance for the other CM types in the general population [18], except for two recent studies in Germany that found more severe CEA, in addition to CSA, in women than in men [15,16]. Finally, relationships between participants’ age and self-reported CM have recently been identified, with older age being significantly associated with a higher prevalence of CPN [15], and older birth cohorts being associated with higher rates of any CM, particularly childhood neglect [16].

It has been well established that individuals with MDD or BD have a higher CM burden than the general population. Although prevalence estimates can vary among studies [19], the extremely high CM rate in MDD and BD is noted to be a consistent finding, reaching up to 50% of individuals in both diagnostic groups who reported at least one CM type [7,19,20]. Exposure to CM has a negative impact on the course of the disorders, as it is associated with an increased risk in MDD or BD first-onset, earlier onset, more severe illness, and poor treatment response [7,21,22]. 

The potential moderating effects of sex on the relationships between CM and MDD or BD are being investigated. There are sexual dimorphisms in neural, hormonal, and immune systems and functions, which may affect sex differences in the short- and long-term consequences in response to environmental stressors [23,24]. Furthermore, sex may influence the prevalence and nature of CM, and different CM types may interact differently with sex [7,24]. However, it remains unclear to what extent the interplay between sex and CM influences the higher MDD risk in women than in men [25], or the sex differences in both MDD [26,27] and BD [28,29] clinical features and courses. Finally, the sex differences in CM prevalence among patients with MDD or BD remain obscure. 

A recent meta-analysis demonstrated that associations between CM and MDD in adulthood were stronger in women than in men, but there was insufficient evidence to conclusively identify sex differences in the effects of CM on MDD development [30]. Similarly, a subsequent meta-analysis has failed to find any sex differences in the association between CM and MDD morbidity or in the severity of depressive symptoms in adulthood [31]. In contrast, a recent study revealed a synergistic effect of female sex and unfavorable childhood experiences on the 12-month prevalence of major depressive episodes in US adults [32]. Similarly, a Brazilian population-based study identified CM as a significant risk factor for MDD in women rather than in men [33]. In a sample of Spanish outpatients with MDD, only women showed an association between CM burden and suicide attempts [34]. Additionally, in a sample of Italian patients with severe mental disorders, including BD and MDD, patients with moderate to severe CM were found to be more likely to be female and to have more suicidal tendencies [35]. In samples of both French and Norwegian inpatients and outpatients with BD [36], as well as in Italian outpatients with BD [37], women reported a CM history more frequently than men. However, the former study [36] found that women with CM had more severe illness than men with CM, whereas the latter study [37] failed to confirm this finding. 

Lastly, a recent meta-analysis including CM prevalence studies in patients with MDD or BD up to 2019 revealed that there was a higher CSA prevalence among females than males in patients with MDD, with no sex differences reported for any CM type in patients with BD [19]. 

In general, the heterogeneity of methodology and outcomes among studies has been found to be substantial, and epidemiologic data on the sex-based CM prevalence in MDD and BD are extremely scarce to lacking in some countries, including Italy [19]. Therefore, further research on sex differences is warranted [19,21]. 

To the best of our knowledge, other than the aforementioned study in outpatients with BD [37], no Italian studies on this topic have been reported. 

In order to fill this gap, we aimed to investigate whether there are any sex differences in the scope of self-reported CM in a large sample of Italian adult inpatients suffering from MDD or BD. CM was assessed using the Childhood Trauma Questionnaire (CTQ), which is the most widely used tool for retrospective CM assessment for clinical and research purposes [10,19,38]. As relationships between individuals’ age and CTQ-based self-reported CM in the general population were found [15,16], the potential impacts of patients’ age on the CM self-report have been investigated.

## 2. Materials and Methods

### 2.1. Study Design

This retrospective, observational study used the data from patients’ electronic medical records (EMRs) at Villa San Benedetto Menni Hospital, Albese con Cassano, Como, Italy.

### 2.2. Participants

In total, 503 adult (age ≥ 18 years) patients with a primary diagnosis of MDD (*n* = 335; women, *n* = 255; men, *n* = 80) or BD (type I/II/unspecified; *n* = 168; women, *n* = 97; men, *n* = 71) according to the Diagnostic and Statistical Manual of Mental Disorders (DSM-5) [39], and being in a major depressive episode (DSM-5 criteria), and hospitalized for a 4-week psychiatric rehabilitation program, were included in this study. 

The hospital’s inpatients came from various clinical centers in Italy, primarily from northern Italy, and were usually referred by their high-contact psychiatrists. The standardized hospitalization criteria for the rehabilitation program included the following: being 18 years or older; being at low risk for suicide, as clinically assessed by the high-contact psychiatrists immediately before the hospitalization and confirmed by the hospital’s clinicians at the beginning of the hospitalization; being completely self-sufficient; and, for patients with BD, being in a major depressive episode. 

This study is a sub-analysis of data collected from a larger ongoing longitudinal observational study carried out at Villa San Benedetto Menni Hospital entitled “Effects of treatments on quality of life of patients with psychiatric disorders with or without medical comorbidity.” This entire observational study adheres to the principles of the Helsinki Declaration, and has been approved by the Ethical Committee of the Local Health Authority of the Province of Como, Italy (Protocol Number: 0021453; first approval: 1 July 2010, with subsequent four-year renewals until July 2022). At the time of hospitalization, all inpatients were asked to voluntarily provide written informed consent to use their clinical data collected during hospitalization for the purpose of the entire aforementioned study. 

The inpatients hospitalized between January 2014 and December 2019 were retrospectively selected using data from their EMRs. During the reference period of the presented results, approximately 70% of inpatients gave their written informed consent to participate in the entire aforementioned study. No other inclusion criteria for this sub-analysis study were used apart from the date and the aforementioned written informed consent, diagnoses, and hospital admission criteria. The patients were selected using the following exclusion criteria: a history of psychotic disorders; mood disorders due to another medical condition; suspected or diagnosed (intelligence quotient < 70) mental retardation; dementia; and any neurological disease or medical condition that would affect the reliability of the self-administered assessment. In cases of uncertainty about patient selection, a consensus between the investigators and the psychiatrist who had cared for the patient during their hospitalization was reached. When a patient was hospitalized more than once, the first admission was only considered.

### 2.3. Procedures and Measures

In this study, only EMR variables collected by clinicians within the first 3 days of patient admission prior to any pharmaceutical modifications and at the start of the rehabilitation program were used. Each EMR included a wide range of historical and clinical variables that psychiatrists, psychologists, and nurses collected during hospitalization following standardized procedures that are part of Villa San Benedetto Menni Hospital’s standard clinical practice to ensure homogeneous clinical assessment and data collection for all inpatients. For this study, the following variables were considered for each patient: (1) socio-demographic variables such as age, sex, and years of education; (2) psychiatric disorder-related variables such as illness severity at the time of admission, as measured by clinician-administered Clinical Global Impression–Severity (CGI–S) scale scores ranging from 1 (normal/not ill at all) to 7 (among the most severely ill patients) [40], psychotropic medications at the time of admission, and history of psychiatric disorders other than MDD or BD (classified as “*no*” versus “*yes*”); and (3) retrospective self-report CM variables, as assessed by the CTQ short-form, which is a 28-item self-report inventory that measures CM occurrence before the age of 18. The CTQ presents good internal consistency, test–retest reliability, and convergence validity when compared to CM reports from other sources, including direct interviews [10,38]. The CTQ assesses five types of maltreatment, referred to as childhood trauma(s) [CT(s)], namely emotional, physical, and sexual abuse (CEA, CPA, CSA, respectively), and emotional and physical neglect (CEN and CPN, respectively), with five items representing each type. Individuals respond to a series of statements concerning childhood events, which they rate on a 5-point Likert scale based on their frequency. The response options are “*never true*” (scored as 1), “*rarely true*” (scored as 2), “*sometimes true*” (scored as 3), “*often true*” (scored as 4), and “*very often true*” (scored as 5). Item scores are summed to produce five subscale scores that quantify the severity of each maltreatment type (scoring range for each CT type: 5–25; the higher the score of each CT type, the higher the exposure level to each CT type); a total CTQ score ranging from 25 to 125 can be calculated (the higher the total CTQ score, the higher the global exposure levels to CTs). In addition, cut scores that classify the severity of exposure to each CT type are provided in order to obtain the following four classes: *none or minimal*, *low to moderate*, *moderate to severe*, *and severe to extreme* (Table 1). Finally, the CTQ includes a 3-item minimization/denial scale, with 1 point given for each item endorsed with a score of 5 (“very often true”), while for each item endorsed with a score of less than 5, 0 points are given. The total score of the minimization/denial scale ranges from 0 to 3, and any score from 1 to 3 suggests that maltreatment may be underreported (false negatives). The original CTQ was translated from English into an Italian version for clinical use in our hospital by an author (AA), and this was blindly back-translated from Italian into English by a second author (SD). The back-translated version was then sent to a native English speaker researcher who was fluent in Italian, who amended any minor errors in the final version. Recently, an Italian version of the CTQ that was validated on college students has been published [41]. However, since our study had begun earlier in 2014, we opted to maintain our CTQ version in order to avoid methodological bias in data collection.

### 2.4. Statistical Analysis

For the purpose of this study, the analyses indicated below were conducted separately on the samples of patients with MDD or BD. 

The Student’s *t*-test was used to compare women and men in terms of mean age in years, mean CGI–S scores, and education. The Fisher’s exact test was used to compare women and men in terms of rates of positive history for psychiatric disorders other than MDD or BD, with the distribution of patients with at least one CT type classified as low to moderate, and with the distribution of patients with at least one CT type classified as moderate to severe or severe to extreme.

Seven separate linear regression models were used to test for associations between independent variables such as age and sex, as well as their interactions, and the following dependent variables: CTQ total score, the CTQ-subscale scores, and the number of CTs to which patients had been exposed, i.e., the number of subscales in which the cut scores identified at least a *low to moderate* level of trauma.

Furthermore, six separate logistic regression models were used to test for associations between independent variables such as age and sex, as well as their interactions, and the following dependent variables: exposure to each CT type classified as “*no*” (*none to minimal* exposure severity to that CT type as identified by the cut score) versus “*yes*” (at least a *low to moderate* exposure severity to that CT type as identified by the cut score). The same logistic regression model was applied to the CT minimization/denial scale as a dependent variable classified as “*no*” (no minimization items scored 1) versus “*yes*” (at least one minimization item scored 1). Before performing all the regression analyses, age and sex were mean centered.

In addition, the entire sample of patients with MDD or BD was compared in terms of their socio-demographic and clinical characteristics, CTQ scores, and exposure to each CT type as identified by the cut scores, using the Student’s *t*-test for continuous variables, the Fisher’s exact test for categorical variables, or the Mann–Whitney W test for ordinal variables (Supplementary Materials Table S1). 

As multiple statistical tests were conducted in this study, the significance level (α) was lowered from 0.05 to 0.01 in order to reduce the possibility of obtaining type I errors. Statistical analyses were conducted using the R programming language, version 3.6.3 (R Core Team, R: A Language and Environment for Statistical Computing, R Foundation for Statistical Computing, Vienna, Austria, 2020).

## 3. Results

The descriptive statistics for the two entire samples of patients with MDD or BD are shown in the Supplementary Materials (Table S1). Except for a higher number of women in the MDD sample, no other statistically significant difference was noted in terms of socio-demographic and clinical characteristics or CTQ-related variables between the entire sample of patients with MDD and that with BD (Table S1), 

The descriptive statistics of women and men in the two samples of patients with MDD or BD are shown in Table 1. All patients were given psychotropic medications, including antidepressants (e.g., selective serotonin reuptake inhibitors, serotonin-norepinephrine reuptake inhibitors, tricyclic antidepressants, and other antidepressants), mood stabilizers, second-generation or typical antipsychotics, and benzodiazepines (data not shown). 

In both samples, no statistically significant sex differences were detected in terms of age (MDD: t = 6.9, *p* = 0.04; BD: t = 1.0, *p* = 0.31), years of education (MDD: t = 0.6, *p* = 0.52; BD: t = 0.7, *p* = 0.45), illness severity (CGI–S scores) (MDD: t = −0.8, *p* = 0.39; BD: t = −0.8, *p* = 0.42), rates of patients with positive history of psychiatric disorders other than MDD or BD (MDD: *p* = 0.15; BD: *p* = 0.25), distribution of patients with at least one type of low to moderate CT (MDD: *p* = 0.49; BD: *p* = 0.56), or distribution of patients with at least one type of moderate to severe or severe to extreme CT (MDD: *p* = 0.7; BD: *p* = 0.53). 

In both samples, no significant association was detected between sex and the participants’ age–sex interaction, CTQ total and subscale scores, the number of CTs to which patients had been exposed, the exposure (no versus yes, i.e., at least a low to moderate exposure severity) to each CT type, or CT minimization (Table 2, Table 3, Table 4 and Table 5). 

In contrast, a significant inverse association was detected in both samples between participants’ age and the number of CTs to which patients had been exposed, CTQ total score, CEA subscale score, and the exposure (no versus yes, i.e., at least a low to moderate exposure severity) to CEA (i.e., the older the age, the lower the CTs) (Table 2, Table 3, Table 4 and Table 5). Furthermore, there was a significant inverse association between participants’ age and CSA subscale score in the sample of patients with BD alone (Table 4).

## 4. Discussion

Herein, we present findings from a retrospective study that investigated whether there are sex differences in the self-reported CM, as assessed by the 28-item CTQ, in two large samples of Italian adult patients with MDD or BD who were hospitalized for a 4-week psychiatric rehabilitation program. In addition, we investigated the potential effects of patients’ age on the self-reported CM. Furthermore, we reported the CM distribution in the two entire samples of patients with MDD or BD, which can be useful for future epidemiologic meta-analyses. This is the first study of its kind and purpose in Italy. 

In accordance with previous international reports, we identified considerable rates of CM in entire samples of patients with MDD or BD that were higher than those typically reported in the general population [14,15,16]. Furthermore, no significant differences in CM were found between the two samples. There were approximately 58% of patients with MDD and 54% of those with BD who declared at least one type of moderate to severe CT, but the rates were noted to increase to 84% and 80%, respectively, when patients with at least one type of low to moderate CT were considered. When the different types of moderate to severe CTs were investigated, the top two CTs with the highest prevalence among patients with MDD were found to be CEN (42%) and CEA (41%), followed by CPN (23%), CPA (13%), and CSA (6%). Our rates were generally similar to the pooled rates recently established in MDD samples from other European countries, except for our CSA rate, which was lower than the pooled rate estimated at 19% [19]. Likewise, our rates in patients with BD (i.e., CEN, 31%; CEA, 43%; CPN, 19%; CPA, 10%; and CSA, 6%) were found to be similar to the pooled prevalence in BD samples, except for our CSA rate, which was lower than the pooled rate estimated at 19% [19].

In this study, the moderate to severe CSA rates were particularly low. The lack of other Italian studies reporting these rates in patients with MDD or BD makes it difficult to interpret this finding; thus, this obtained result requires further validation in the future. Only two Italian studies, with different goals than ours, reported the mean scores of CTQ-based CSA in outpatients with MDD or BD [42], or in patients with BD [37]. The former study found very low to lower than our CSA mean scores in both MDD and BD, but the latter identified a CSA mean score in BD that was similar to our BD sample’s mean score. It should be noted that in the study by Serafini and co-workers [42], all the CTQ-subscale scores, except for CEA, were unexpectedly low in both MDD and BD samples. However, our findings in patients are consistent with the lower CSA rate in the Italian general population compared to the approximately 13% reported in various international studies [14,15,16]. According to an Italian official estimate, approximately 6% of a large sample of Italian women aged 19 to 60 experienced some form of sexual abuse during childhood [43], while a more recent official report indicated that 5% of Italian adults (7.8% of women and 2.2% of men) were subjected to sexual acts against their will when they were under 18 [44]. Among maltreated Italian children, SA appeared to be less prevalent than other forms of maltreatment as well as less prevalent than among maltreated children in other European countries [13,45]. It remains unclear to what extent these lower CSA rates reflect a real reduced frequency of occurrence or are attributable to greater difficulty in identifying, disclosing, or reporting this form of abuse in Italy. Finally, it should be noted that an additional 8% of our patients with MDD and 9% of those with BD reported mild CSA forms (i.e., *low to moderate* exposure severity), reaching total percentages of patients with CSA of 14% and 15%, respectively. Although the rates of maltreated patients in other studies [19,35] did not include individuals with mild CT forms, it cannot be excluded that a less severe CSA may be of significance to psychopathology. 

In both MDD and BD samples, no statistically significant sex differences were detected in a variety of CM aspects examined, including the following: the rates of patients with at least one type of low to moderate CT or moderate to severe CT; the CTQ total and subscale mean scores; the mean number of CTs to which patients had been exposed; and having suffered at least a low to moderate exposure severity level to each CT type (*no* versus *yes*). In addition, the minimization/denial scale (MDS) scores did not differ between women and men, indicating no significant impact of sex on the possibility that maltreatment was underreported. These scores are infrequently reported, and MDS validation studies are often scarce. However, the rates of potential underreporting in our samples were relatively low and similar to those established in a large sample of patients with severe mental disorders, including MDD and BD, who showed lower minimization levels than healthy individuals [46]. 

The lack of significant CM sex differences in our samples is mostly consistent with those documented in other countries [19]. In contrast, we were unable to detect significantly higher CSA rates in female patients with MDD than in males, which was the only significant sex-based difference identified in the most recent meta-analysis [19]. However, although our MDD sample size is large, we were unable to rule out type II errors, and further Italian studies are warranted to confirm our negative CSA findings.

It should be noted that there is a tendency toward higher CSA scores in female patients with BD than in males, as well as a higher rate of female patients who were subjected to at least a low to moderate CSA (20% versus 8%, respectively), although not statistically significant. This trend is consistent with the greater CSA burden in Italian women with BD than in men, which was recently found [37]. We adopted a conservative level of significance in order to reduce the type I error risk associated with large numbers of statistical comparisons, at the expense of increasing the type II error risk. Therefore, further studies in larger BD samples are needed in order to evaluate whether this tendency of higher CSA among female patients can be confirmed statistically. 

Overall, our findings do not support the notion that sex substantially influences the prevalence and type of self-reported CM, at least as assessed by the CTQ. Female and male patients with MDD or BD appeared to have a great burden of different CM types, with no significant sex differences. These results suggest that the difference in the impact of CM on the MDD or BD clinical course between women and men [33,34,35,36] may be due to sexual dimorphism in terms of neural, hormonal, and immune systems and functions, which may influence sex-based differential responses to CM [23,24], rather than sex differences in the burden of CM per se. 

In this study, we detected for the first time a significant association between increasing patients’ age and a decreasing burden of some CM aspects, including the following: CTQ total score, number of CTs to which patients had been exposed, and CEA-related burden, in both samples, with no significant sex differences. Furthermore, in patients with BD, this association was also found for the CSA subscale score. This pattern differs from that recently found in the German general population, where older age was associated with higher CTQ-based CPN rates [15], and older birth cohorts were associated with higher rates of any CM, particularly childhood neglect [16], most likely due to unfavorable socioeconomic conditions and different social norms during the childhood time of older generations [15]. Our contradictory findings could be attributed to the burden of psychopathology on cognitive functions and recall. Our samples had an elderly mean age, and patients admitted to Villa San Benedetto Menni Hospital for a psychiatric rehabilitation program typically have chronic psychiatric conditions of long durations. Therefore, it is conceivable that the well-documented cognitive impairment associated with MDD and BD may have compromised retrospective recall of CM, particularly in older patients [47,48,49]. Unfortunately, the sample size did not allow us to analyze the CM distribution across age cohorts in order to achieve a more comprehensive understanding of this topic. 

## 5. Limitations

Aside from the limitations previously noted, this study had several others. The CM data were collected through a retrospective self-reported measure of CTs. Although retrospective data have often been found to be reliable and stable in psychiatric populations [46], we cannot rule out the possibility that recall bias, illness representation, or mood effects in patients who were hospitalized and depressed at the time of self-reporting may have confounded the retrospective evaluation of CM in our samples. 

Our samples had an elderly mean age, and patients were admitted in order to undergo a psychiatric rehabilitation program. Therefore, future studies with samples in different settings are required to understand whether our findings can be confirmed and generalized. Furthermore, we cannot exclude that the relatively lower number of male patients may have influenced the results. 

There are certain methodological concerns with regard to the CTQ. While confirmatory factor analyses confirmed that the 5-factor solution was the model that best fit Italian data from nonclinical samples [41], exploratory factor analysis in a heterogeneous sample of Italian psychiatric patients found that a 3-factor solution (emotional neglect/abuse, sexual abuse, physical neglect/abuse) was the most appropriate, implying the CTQ’s possible lack of structural invariance in cross-cultural adaptations [50]. Furthermore, since there are no Italian studies to validate the use of specific cut scores for CT severity categories, we used the defined cut scores from the original studies [10,38]. Finally, we were unable to provide direct comparisons with the CM background acquired from our patient samples due to a lack of CM data from matched samples of healthy individuals. Overall, while using the original CTQ factors and scoring allows us to compare our results to those of other international studies, we cannot rule out the possibility that they are insufficient for screening CM in Italian samples. 

The CTQ does not collect data about crucial CM features such as perpetrator(s), age and duration of CM exposure, and CT chronicity or recurrence. These factors may exhibit sex differences [51] and may be involved in sex-based different outcomes in response to CM. Furthermore, we did not explore many other types of adverse childhood events which may exhibit sex differences and influence sexual variations in psychopathological outcomes after adversities.

## 6. Conclusions

In conclusion, despite these limitations, this study revealed that both women and men with MDD or BD experienced a similar, considerable CM burden, with no significant sex differences. 

Due to the pernicious effects of CM on the course of both disorders and treatment responses [7,21,22], our findings support the importance of evaluating CM in both female and male patients with MDD and BD in clinical practice, in order to identify patients who may require more careful monitoring and special therapeutic attention. 

Many studies have emphasized the need for CM-informed interventions in psychiatry and a more personalized approach to treatment guided by factors, such as CM, that contribute to poor response to therapies. Although evidence remains limited, recent preliminary studies suggest that venlafaxine-XR, escitalopram, or antidepressants with high affinity for serotonin transporters may be more appropriate than other compounds for patients with MDD and a childhood abuse history [52]; maltreated patients with MDD or BD appeared to benefit from vortioxetine [53] or infliximab, respectively [54]. 

In this context, including the CM assessment as part of the routine psychiatric clinical practice for both female and male patients with MDD or BD should be considered. 

## Figures and Tables

**Table 1:** Descriptive statistics of women and men in the sample of patients with MDD or BD.

	Patients with MDD (*n* = 335)				Patients with BD (*n* = 168)			
	Women (*n* = 255; 76%)		Men (*n* = 80; 24%)		Women (*n* = 97; 58%)		Men (*n* = 71; 42%)	
Characteristics *	*Mean*/N	*SD*/%	*Mean*/N	*SD*/%	*Mean*/N	*SD*/%	*Mean*/N	*SD*/%
Age, years	*64.9*	*12.06*	*61.9*	*11.2*	*62.6*	*12.7*	*60.6*	*13.1*
Age, range	28–89		30–85		29–85		39–90	
Education, years	*10.5*	*4.41*	*10.9*	*3.3*	*11.4*	*4.0*	*11.0*	*3.3*
Clinical Global Impression Scale-Severity (CGI-S)	*3.4*	*0.9*	*3.5*	*0.8*	*3.6*	*0.8*	*3.7*	*0.8*
History of psychiatric disorders other than MDD/BD *(yes)*	100	39%	39	49%	28	29%	27	38%
Childhood Trauma Questionnaire (CTQ)								
Scores								
Sexual abuse	*6.6*	*3.8*	*5.9*	*2.4*	*6.7*	*3.5*	*5.8*	*1.8*
Physical abuse	*6.6*	*3.5*	*7.0*	*4.4*	*6.1*	*2.5*	*6.8*	*3.5*
Emotional abuse	*8.7*	*4.9*	*8.1*	*4.6*	*9.1*	*5.1*	*7.9*	*4.1*
Physical neglect	*8.0*	*3.3*	*7.8*	*3.2*	*7.6*	*3.3*	*7.3*	*2.7*
Emotional neglect	*13.6*	*6.1*	*13.1*	*5.4*	*12.1*	*5.9*	*12.3*	*4.5*
Total score	*43.5*	*17.0*	*41.8*	*16.0*	*41.5*	*15.8*	*40.1*	*13.2*
Number of CTs to which patients had been exposed **	*1.9*	*1.5*	*2.1*	*1.4*	*2.0*	*1.6*	*1.9*	*1.4*
Classification ***								
*Sexual abuse (SA)*								
None (or Minimal) (SA subscale score = 5)	214	84%	71	89%	77	79%	65	92%
Low (to Moderate) (SA subscale score = 6–7)	22	9%	6	8%	10	10%	5	7%
Moderate (to Severe) (SA subscale score = 8–12)	5	2%	2	3%	6	6%	1	1%
Severe (to Extreme) (SA subscale score ≥ 13)	14	5%	1	1%	4	4%	0	0%
*Physical abuse (PA)*								
None (or Minimal) (PA subscale score = 5–7)	208	82%	67	84%	80	82%	57	80%
Low (to Moderate) (PA subscale score = 8–9)	16	6%	1	1%	9	9%	5	7%
Moderate (to Severe) (PA subscale score = 10–12)	14	5%	3	4%	5	5%	3	4%
Severe (to Extreme) (PA subscale score ≥ 13)	17	7%	9	11%	3	3%	6	8%
*Emotional abuse (EA)*								
None (or Minimal) (EA subscale score = 5–8)	104	41%	37	46%	37	38%	30	42%
Low (to Moderate) (EA subscale score = 9–12)	42	16%	14	18%	15	15%	13	18%
Moderate (to Severe) (EA subscale score = 13–15)	66	26%	15	19%	24	25%	20	28%
Severe (to Extreme) (EA subscale score ≥ 16)	43	17%	14	18%	21	22%	8	11%
*Physical neglect (PN)*								
None (or Minimal) (PN subscale score = 5–7)	147	58%	48	60%	61	63%	49	69%
Low (to Moderate) (PN subscale score = 8–9)	48	19%	15	19%	17	18%	8	11%
Moderate (to Severe) (PN subscale score = 10–12)	32	13%	9	11%	10	10%	9	13%
Severe (to Extreme) (PN subscale score ≥ 13)	28	11%	8	10%	9	9%	5	7%
*Emotional neglect (EN)*								
None (or Minimal) (EN subscale score = 5–9)	72	28%	24	30%	40	41%	18	25%
Low (to Moderate) (EN subscale score = 10–14)	75	29%	24	30%	23	24%	36	51%
Moderate (to Severe) (EN subscale score = 15–17)	32	13%	15	19%	15	15%	6	8%
Severe (to Extreme) (EN subscale score ≥ 18)	76	30%	17	21%	19	20%	11	15%
Patients with at least one type of CT classified as Low (to Moderate)	216	85%	65	81%	76	78%	59	83%
Patients with at least one type of CT classified as Moderate (to Severe) or Severe (to Extreme)	150	59%	45	56%	54	56%	36	51%
Minimization/denial								
No items scored 1	183	72%	55	69%	66	68%	55	77%
One item scored 1	47	18%	15	19%	15	15%	10	14%
Two items scored 1	14	5%	7	9%	10	10%	5	7%
Three items scored 1	11	4%	3	4%	6	6%	1	1%
At least one item scored 1	72	28%	25	31%	31	32%	16	23%

* The “Characteristics” are expressed as number (N) and % or mean and standard deviation (SD): the characteristics expressed as mean and SD are *italicized*; BD: bipolar disorder; CT(s): childhood trauma(s); MDD: major depressive disorder; ** the number of subscales in which the cut scores identified at least a low to moderate level of trauma; *** for each type of childhood trauma, the exposure severity is classified based on the cut scores of each subscale reported in the brackets.

**Table 2:** Linear regression analysis in patients with MDD.

	Patients with MDD (*n* = 335)																		
	Sex						Age						Interaction Sex–Age						
	B	SE	95% Confidence Interval for B (Lower Bound)	95% Confidence Interval for B (Upper Bound)	t	*p*-Value	B	SE	95% Confidence Interval for B (Lower Bound)	95% Confidence Interval for B (Upper Bound)	t	*p*-Value	B	SE	95% Confidence Interval for B (Lower Bound)	95% Confidence Interval for B (Upper Bound)	t	*p*-Value	Adjusted R^2^
**CTQ Scores**																			
Sexual abuse	−0.571	0.477	−1.809	0.666	−1.197	0.232	−0.031	0.017	−0.075	0.013	−1.843	0.066	0.009	0.044	−0.104	0.122	0.204	0.839	0.006
Physical abuse	0.534	0.500	−0.764	1.831	1.066	0.287	−0.031	0.018	−0.077	0.015	−1.753	0.081	0.014	0.046	−0.105	0.132	0.297	0.767	0.006
Emotional abuse	−0.531	0.661	−2.247	1.184	−0.804	0.422	−0.116	0.023	−0.176	−0.055	−4.924	**<0.001**	0.001	0.060	−0.155	0.158	0.024	0.981	0.071
Physical neglect	0.058	0.453	−1.118	1.233	0.127	0.899	0.002	0.016	−0.040	0.043	0.106	0.915	−0.033	0.041	−0.140	0.074	−0.801	0.424	−0.008
Emotional neglect	−0.300	0.853	−2.513	1.913	−0.352	0.725	−0.051	0.030	−0.130	0.027	−1.695	0.091	−0.025	0.078	−0.227	0.176	−0.327	0.744	0.000
Total score	−0.809	2.315	−6.812	5.193	−0.350	0.727	−0.229	0.082	−0.442	−0.016	−2.785	**0.006**	−0.033	0.211	−0.581	0.515	−0.157	0.876	0.016
Number of CTs to which patients had been exposed *	−0.166	0.203	−0.692	0.360	−0.817	0.415	−0.021	0.007	−0.039	−0.002	−2.851	**0.005**	−0.003	0.019	−0.051	0.045	−0.184	0.854	0.019

CTQ: Childhood Trauma Questionnaire; CT(s): childhood trauma(s); MDD: major depressive disorder; N: number of patients; SE: standard error of the regression coefficient; * the number of subscales in which the cut scores identified at least a low level of trauma; *p*-values considered significant (i.e., <0.01) are bolded.

**Table 3:** Logistic regression analysis in patients with MDD.

Patients with MDD (*n* = 335)																					
	Sex							Age							Interaction Sex–Age						
	OR	B	SE	95% Confidence Interval for B (Lower Bound)	95% Confidence Interval for B (Upper Bound)	t	*p*-Value	OR	B	SE	95% Confidence Interval for B (Lower Bound)	95% Confidence Interval for B (Upper Bound)	t	*p*-Value	OR	B	SE	95% Confidence Interval for B (Lower Bound)	95% Confidence Interval for B (Upper Bound)	t	*p*-Value
CTQ Classification *																					
Sexual abuse	0.800	−0.223	0.422	−1.448	0.788	−0.529	0.597	0.980	−0.020	0.014	−0.056	0.016	−1.406	0.160	0.992	−0.008	0.037	−0.108	0.089	−0.209	0.834
Physical abuse	0.972	−0.028	0.402	−1.159	0.955	−0.071	0.944	0.964	−0.036	0.014	−0.074	0.000	−2.561	0.010	1.032	0.031	0.036	−0.063	0.126	0.870	0.384
Emotional abuse	0.689	−0.372	0.292	−1.132	0.380	−1.275	0.202	0.954	−0.048	0.011	−0.077	−0.020	−4.302	**<0.001**	1.004	0.004	0.028	−0.070	0.074	0.160	0.873
Physical neglect	0.942	−0.060	0.290	−0.825	0.678	−0.207	0.836	1.004	0.004	0.010	−0.022	0.031	0.421	0.674	0.980	−0.020	0.026	−0.090	0.048	−0.769	0.442
Emotional neglect	0.897	−0.109	0.304	−0.880	0.700	−0.357	0.721	0.984	−0.016	0.011	−0.045	0.012	−1.423	0.155	0.988	−0.012	0.028	−0.088	0.060	−0.412	0.680
**Minimization/** denial **	1.347	0.298	0.299	−0.492	1.061	0.996	0.319	1.004	0.004	0.011	−0.024	0.033	0.396	0.692	1.019	0.019	0.028	−0.052	0.093	0.691	0.490

CTQ: Childhood Trauma Questionnaire; CT(s): childhood trauma(s); MDD: major depressive disorder; N: number of patients; OR: odds ratio; SE: standard error of the regression coefficient; * the exposure to each type of CT is classified as “*no*” (none to minimal exposure severity to that type of CT as identified by the cut score) versus “*yes*” (at least a low to moderate level of exposure severity to that type of CT trauma as identified by the cut score); ** minimization/denial is classified as “*no*” (no minimization items scored 1) versus “*yes*” (at least one minimization item scored 1); *p*-values considered significant (i.e., <0.01) are bolded.

**Table 4:** Linear regression analysis in patients with BD.

	Patients with BD (*n* = 168)																		
	Sex						Age						Interaction Sex–Age						
	B	SE	95% Confidence Interval for B (Lower Bound)	95% Confidence Interval for B (Upper Bound)	t	*p*-Value	B	SE	95% Confidence Interval for B (Lower Bound)	95% Confidence Interval for B (Upper Bound)	t	*p*-Value	B	SE	95% Confidence Interval for B (Lower Bound)	95% Confidence Interval for B (Upper Bound)	t	*p*-Value	Adjusted R^2^
**CTQ Scores**																			
Sexual abuse	−1.052	0.487	−2.324	0.219	−2.163	0.032	−0.056	0.019	−0.105	−0.007	−2.959	**0.004**	0.069	0.038	−0.030	0.167	1.829	0.070	0.081
Physical abuse	0.547	0.494	−0.744	1.839	1.107	0.270	−0.048	0.019	−0.098	0.002	−2.523	0.013	−0.029	0.038	−0.129	0.071	−0.769	0.443	0.042
Emotional abuse	−1.411	0.811	−3.531	0.708	−1.739	0.084	−0.131	0.031	−0.213	−0.049	−4.174	**<0.001**	0.055	0.063	−0.109	0.219	0.876	0.382	0.108
Physical neglect	−0.290	0.553	−1.735	1.156	−0.524	0.601	−0.003	0.021	−0.059	0.053	−0.147	0.883	0.073	0.043	−0.039	0.185	1.709	0.090	0.002
Emotional neglect	−0.516	0.924	−2.931	1.898	−0.559	0.577	−0.082	0.036	−0.175	0.012	−2.277	0.024	0.112	0.072	−0.075	0.299	1.570	0.119	0.030
Total score	−2.614	2.545	−9.262	4.034	−1.027	0.306	−0.321	0.099	−0.579	−0.064	−3.257	**0.001**	0.282	0.197	−0.233	0.796	1.431	0.155	0.064
Number of CTs to which patients had been exposed *	−0.181	0.258	−0.855	0.493	−0.702	0.484	−0.028	0.010	−0.054	−0.002	−2.795	**0.006**	0.022	0.020	−0.030	0.074	1.112	0.268	0.040

CTQ: Childhood Trauma Questionnaire; BD: bipolar disorder; CT(s): childhood trauma(s); N: number of patients; SE: standard error of the regression coefficient; * the number of subscales in which the cut scores identified at least a low level of trauma; *p*-values considered significant (i.e., <0.01) are bolded.

**Table 5:** Logistic regression analysis in patients with BD.

Patients with BD (*n* = 168)																					
	Sex							Age							Interaction Sex–Age						
	OR	B	SE	95% Confidence Interval for B (Lower Bound)	95% Confidence Interval for B (Upper Bound)	t	*p*-Value	OR	B	SE	95% Confidence Interval for B (Lower Bound)	95% Confidence Interval for B (Upper Bound)	t	*p*-Value	OR	B	SE	95% Confidence Interval for B (Lower Bound)	95% Confidence Interval for B (Upper Bound)	t	*p*-Value
CTQ Classification *																					
Sexual abuse	0.397	−0.924	0.512	−2.393	0.325	−1.803	0.071	0.974	−0.026	0.019	−0.075	0.023	−1.393	0.164	1.042	0.041	0.039	−0.058	0.147	1.060	0.289
Physical abuse	1.103	0.098	0.459	−1.149	1.267	0.214	0.830	0.964	−0.036	0.017	−0.082	0.007	−2.113	0.035	0.977	−0.023	0.034	−0.115	0.064	−0.683	0.495
Emotional abuse	0.760	−0.275	0.375	−1.253	0.695	−0.732	0.464	0.945	−0.057	0.016	−0.101	−0.018	−3.561	**<0.001**	1.010	0.010	0.032	−0.075	0.092	0.311	0.756
Physical neglect	0.707	−0.347	0.370	−1.330	0.593	−0.938	0.348	1.014	0.014	0.015	−0.023	0.054	0.972	0.331	1.057	0.056	0.030	−0.020	0.139	1.837	0.066
Emotional neglect	1.193	0.176	0.383	−0.809	1.182	0.461	0.645	0.962	−0.039	0.016	−0.082	−0.001	−2.516	0.012	1.029	0.029	0.031	−0.052	0.109	0.926	0.355
**Minimization/** denial **	0.918	−0.085	0.429	−1.219	1.030	−0.199	0.842	1.031	0.031	0.017	−0.012	0.078	1.806	0.071	0.927	−0.076	0.034	−0.167	0.009	−2.268	0.023

CTQ: Childhood Trauma Questionnaire; BD: bipolar disorder; CT(s): childhood trauma(s); N: number of patients; OR: odds ratio; SE: standard error of the regression coefficient; * the exposure to each type of CT is classified as “*no*” (none to minimal exposure severity to that type of CT as identified by the cut score) versus “*yes*” (at least a low to moderate level of exposure severity to that type of CT trauma as identified by the cut score); ** minimization/denial is classified as “*no*” (no minimization items scored 1) versus “*yes*” (at least one minimization item scored 1); *p*-values considered significant (i.e., <0.01) are **bolded**.

## Data Availability

The data supporting the presented results are available on request from the corresponding author. The data are not publicly available to protect the privacy of the patients.

## Supplementary Materials

The following supporting information can be downloaded at: https://www.mdpi.com/article/10.3390/brainsci12060804/s1, Table S1: Descriptive statistics and comparisons of the two entire samples.
