# Supplementary material for: No Sex Differences in Self-Reported Childhood Maltreatment in Major Depressive and Bipolar Disorders: A Retrospective Study
Source: Brain Sci. 2022 Jun 19;12(6):804. doi: 10.3390/brainsci12060804 (PMC9220896; doi:10.3390/brainsci12060804)
Supplement: Supplementary file 1 [file brainsci-12-00804-s001.zip › brainsci-1769452-supplementary.pdf]

Table S1. **Descriptive statistics and comparisons of the two entire samples**

Characteristics*	Patients with MDD (N= 335) *Mean*/N	*SD*/%	Patients with BD (N= 168) *Mean*/N	*SD*/%	T-Test§ / Fisher's exact† /Mann-Whitney (W)	p-value
**Age, years**	*64.2*	*11.9*	*61.8*	*12.9*	2.0§	0.05
**Age, range**	28-89		29-90			
**Sex, women**	255	76%	97	58%	_†	**<.001**
**Education, years**	*10.6*	*4.2*	*11.3*	*3.7*	1.8§	0.08
**Clinical Global Impression Scale-Severity (CGI-S)**	*3.4*	*0.8*	*3.6*	*0.8*	-2.2§	0.03
**History of psychiatric disorders other than MDD/BD *(yes)***	139	41%	55	33%	_†	0.07
**Childhood Trauma Questionnaire (CTQ)**						
**Scores**						
Sexual abuse	*6.5*	*3.5*	*6.3*	*3.0*	0.5§	0.61
Physical abuse	*6.7*	*3.7*	*6.4*	*3.0*	0.9§	0.36
Emotional abuse	*8.6*	*4.8*	*8.6*	*4.7*	-0.0§	0.97
Physical neglect	*8.0*	*3.3*	*7.5*	*3.1*	1.6§	0.11
Emotional neglect	*13.5*	*6.0*	*12.2*	*5.3*	2.4§	0.02
Total score	*43.1*	*16.8*	*40.9*	*14.7*	1.5§	0.13
Number of CTs to which patients had been exposed**	*2.0*	*1.4*	*1.9*	*1.5*	0.7§	0.48
**Classification*** **						
Sexual abuse (SA)						
None or Minimal (SA subscale score= 5)	285	85%	142	85%		
Low to Moderate (SA subscale score= 6-7)	28	8%	15	9%		
Moderate to Severe (SA subscale score= 8-12)	7	2%	7	4%		
Severe to Extreme (SA subscale score≥13)	15	4%	4	2%	28034.5	0.91
Physical abuse (PA)						
None or Minimal (PA subscale score= 5-7)	275	82%	137	82%		
Low to Moderate (PA subscale score= 8-9)	17	5%	14	8%		
Moderate to Severe (PA subscale score= 10-12)	17	5%	8	5%		
Severe to Extreme (PA subscale score≥13)	26	8%	9	5%	28171.5	0.98
Emotional abuse (EA)						
None or Minimal (EA subscale score= 5-8)	141	42%	67	40%		

Low to Moderate (EA subscale score= 9-12)	56	17%	28	17%		
Moderate to Severe (EA subscale score= 13-15)	81	24%	44	26%		
Severe to Extreme (EA subscale score≥16)	57	17%	29	17%	27486	0.65
Physical neglect (PN)						
None or Minimal (PN subscale score= 5-7)	195	58%	110	65%		
Low to Moderate (PN subscale score= 8-9)	63	19%	25	15%		
Moderate to Severe (PN subscale score= 10-12)	41	12%	19	11%		
Severe to Extreme (PN subscale score≥13)	36	11%	14	8%	30163	0.13
Emotional neglect (EN)						
None or Minimal (EN subscale score= 5-9)	96	29%	58	35%		
Low to Moderate (EN subscale score= 10-14)	99	30%	59	35%		
Moderate to Severe (EN subscale score= 15-17)	47	14%	21	13%		
Severe to Extreme (EN subscale score≥18)	93	28%	30	18%	31668	0.02
**Patients with at least one type of CT classified as Low to Moderate**	281	84%	135	80%	_†	0.32
**Patients with at least one type of CT classified as Moderate to Severe or Severe to Extreme**	195	58%	90	54%	_†	0.34
**Minimization/denial**						
No items scored 1	238	71%	121	72%		
One item scored 1	62	19%	25	15%		
Two items scored 1	21	6%	15	9%		
Three items scored 1	14	4%	7	4%		
At least one item scored 1	97	29%	47	28%	28202.5	0.96

*The "Characteristics" are expressed as number (N) and % or mean and standard deviation (SD): the characteristics expressed as mean and SD are *italicized*; BD: bipolar disorder; CT(s): childhood trauma(s); MDD: major depressive disorder; **the number of sub-scales in which the Cut Scores identified at least a low (to moderate) level of trauma; ***For each type of childhood trauma, the classification of exposure severity is based on the Cut Scores of each subscale reported in the brackets; p-values considered significant (i.e. <0.01) are **bolded**
